# The impact of comorbidities on the all-cause mortality of surgically treated non-small cell lung cancer patients - visualization with the aid of a comorbidome

**DOI:** 10.1186/s12893-025-02995-6

**Published:** 2025-07-04

**Authors:** Julia Zimmermann, Julia Walter, Mircea Gabriel Stoleriu, Julia Kovács, Gökce Yavuz, Fuad Damirov, Niels Reinmuth, Martina Merk, Rudolf A. Hatz, Amanda Tufman, Christian P. Schneider

**Affiliations:** 1 Division of Thoracic Surgery, LMU University Hospital, LMU Munich and Asklepios Lung Clinic Gauting, Munich, Germany; 2 Department of Internal Medicine V, LMU University Hospital, LMU Munich, Germany; 3 Department of Thoracic Oncology, Asklepios Lung Clinic Gauting, Munich, Germany; 4 Comprehensive Pneumology Center Munich, German Center of Lung Research (DZL), Munich, Germany

**Keywords:** Non-small cell lung cancer, Surgery, Comorbidities, Comorbidome, Thoracotomy, Video-assisted thoracoscopic surgery

## Abstract

**Backgrounds:**

Lung cancer patients often have multiple comorbidities. This study aimed to determine which comorbidities had an impact on all-cause mortality in lung cancer patients who had undergone surgical treatment.

**Methods:**

This retrospective study reviewed data from all lung cancer patients who underwent lobectomy or segmentectomy at the Lung Cancer Center Munich between 2011 and 2020. We compared numerical outcomes between patients with minimally invasive surgery and patients with thoracotomy using t-test, and categorical outcomes using Chi2-test or fishers exact test when cell counts were < 6. We used multivariate Cox Regression to model the association between comorbidities and overall survival.

**Results:**

1658 patients (556 minimally invasive,1102 thoracotomy) were included. Across the entire population the comorbidity with the strongest association to death was chronic lymphatic leukemia (HR = 5.15, p = < 0.001), followed by pulmonary fibrosis (HR = 4.06, p = < 0.001), mild liver disease (HR = 2.18, *p* = 0.02), peripheral arterial disease (HR = 1.48, *p* = 0.04) and chronic obstructive pulmonary disease (HR = 1.42, p = < 0.01). In the minimally invasive surgery group chronic lymphatic leukemia was most strongly associated with death (HR = 14.31, *p* = 0.01). This was followed by mild liver disease (HR = 5.01, *p* = 0.01) and myocardial infarction (HR = 2.45, *p* = 0.04). Whereas in the thoracotomy group the strongest associations were fibrosis (HR = 4.20, p = < 0.001) and COPD (HR = 1.51,p = < 0.01).

**Conclusion:**

Most of the comorbidities analyzed do not have a major impact on all-cause mortality after lung surgery. Those that do have a high impact tend to have a very low prevalence.

## Introduction

In 2020 lung cancer was the second most common cancer and the leading cause for cancer death [1]. Surgery, if possible, is known as the first choice of treatment. Lobectomy or segmentectomy are the standard procedures for anatomical resection. They are mainly performed by video-assisted thoracoscopic surgery (VATS) or open surgery (thoracotomy) and should be preferred to any other smaller, non-anatomical resection [2–5]. 

The increase in life expectancy, in conjunction with the fact that lung cancer typically manifests at an advanced age (in Germany the peak age at the initial diagnosis of lung cancer is between 80 and 84 for men, and 65 and 74 for women [6]) suggests the presence of several comorbidities with lung cancer patients. These are likely to be even more common than in other types of cancer [7] and some of them have also been associated with an increased incidence of lung cancer [8, 9]. 

In surgical patients, it is imperative that the treating anesthetist and surgeon, as well as the doctors providing further treatment, be cognizant of any previously documented comorbidities, as these can exert a significant influence on survival, particularly in cancer patients [10]. This principle applies to both the occurrence of events in general and, in particular, in the context of other events, such as an operation. Perioperatively, the cardiovascular system is already subjected to additional stress, which could aggravate further depending on the occurrence of specific comorbidities [11–13]. 

There are limited data on the prognostic value of comorbidities for non-small cell lung cancer (NSCLC) patients undergoing thoracic surgery and on the impact of specific comorbidities on all-cause mortality.

The aim of this study was to analyze a large cohort of patients with non-small cell lung cancer (NSCLC) to determine which specific comorbidities have an impact on all-cause mortality in NSCLC patients undergoing surgery, as well as which comorbidities may have no or negligible effect. Further we wanted to determine whether there are discrepancies between the approaches VATS and thoracotomy. The objective was to enhance awareness of the comorbidities identified in the specific patient population and possibly be able to make perioperative decisions in favor of the patient more quickly and determined. We opted to use a comorbidome to visualize the results graphically. This visualization offers a satisfactory depiction of the relationships between comorbidities and all-cause mortality, as well as their respective prevalences.

## Methods

### Study design, patient cohort and data collection

In this retrospective analysis, we used data of all lung cancer patients undergoing lobectomy or segmentectomy (through thoracotomy or VATS) at the Lung Cancer Center Munich between 2011 and 2020. Preoperatively, all patients were staged according to the current guidelines and discussed at the specific tumor board. We conducted a comprehensive analysis of the comorbidities present in patients with NSCLC who underwent lobectomy or segmentectomy, with a focus on their association with all-cause mortality. The follow-up time was determined as the temporal interval between surgery and date of last contact.

Patients who underwent conversion to thoracotomy were regarded as having undergone a thoracotomy. For patients having had more than one tumor lobectomy or segmentectomy the primary resection was exclusively utilized for analysis purposes. Patients with missing information regarding comorbidities were excluded from the study. The selection of surgical access was contingent on the following criteria. At the beginning of the data analysis, when minimally invasive surgery had not yet become the prevailing standard procedure for lobectomies and segmentectomies, patients with very large tumors or preoperative suspicion of N2 lymph node involvement were predominantly managed through thoracotomy. Patients with smaller tumors and no evidence of lymph node involvement underwent minimally invasive surgery. The advent of increasingly sophisticated minimally invasive surgical techniques the growing expertise of surgeons has engendered a paradigm shift in the management of patients with larger tumors and potential lymph node involvement, who are now able to undergo minimally invasive surgery. All information in the dataset was extracted from electronic patient records and patient archives. This data included information about patient characteristics, namely age at resection, sex and body mass index (BMI) as well as tumor characteristics like tumor size in cm, tumor stage and histological type.

The selection of comorbidities was predominantly facilitated by the Charlson-Comorbidity-Index (CCI). CCI is still of great value for the risk assessment of patients, particularly in the field of lung surgery. In contrast to the standard CCI, the statistics were adjusted to exclude AIDS and rheumatologic disease, as neither of these conditions were present in any of the cohort. All other comorbidities that are also used to calculate the CCI were included, regardless of their prevalence. In view of the fact that the analysis focused on lung cancer patients, a further subdivision of chronic lung diseases had been undertaken, resulting in the categorization of chronic obstructive pulmonary disease (COPD) and fibrosis. The following comorbidities were analyzed: myocardial infarction (MI), diabetes, diabetes with complication, peripheral artery disease (PAD), cerebro vascular disease (CVD), dementia, asthma, peptic ulcer disease, mild liver disease (MLD), hemi-/paraplegia, moderate to severe liver disease, renal insufficiency, chronic lymphatic leukemia, metastatic cancer, coronary heart disease (CAD), atrial fibrillation, arterial hypertension, pulmonary hypertension, fibrosis, New York Heart Association (NYHA), CODP.

Categorization of variables and handling of missing data.

The histological types were categorized into two main types: adenocarcinoma (ACC) and squamous-cell carcinoma (SCC). All remaining histological types were summarized under the category “other histology”.

As BMI-data was missing for several patients, multiple imputation was applied to supplement the missing values.

### Statistical analysis and development of the comorbidome

Patient characteristics are presented as mean values with standard deviation (SD) for numeric variables and absolute and relative frequencies for categorical variables. We compared numerical outcomes between patients with VATS and patients with thoracotomy using t-test. Categorical outcomes were compared using Chi2-test or fishers exact test when cell numbers were < 6. Multivariate Cox Regression was used to model the association between comorbidities and overall survival. All Cox regression models were adjusted by age, sex, tumor size in cm, lymph node involvement (pN), metastases at time of surgery (cM), resection status (R), and histological type. We used hazard ratios (HR) and p-values, as well as prevalences to display our results in a comorbidome. The proportional hazards (PH) assumption was assessed for each covariate and on a global scale using scaled Schoenfeld residuals, applying the Grambsch and Therneau test. In instances where the PH assumption was violated, stratification was employed to account for non-proportionality. To evaluate model discrimination, we calculated the concordance index (C-index). The C-index is a metric that quantifies the model’s capacity to accurately rank survival times.

The created comorbidomes demonstrate the relationship between comorbidities and the all-cause mortality risk of surgically treated lung cancer patients. The radius of the individual comorbidity points is indicative of the prevalence of the comorbidities. The larger the radius, the higher the prevalence. The points within the comorbidity circle had a significant influence on all-cause mortality. The closer they were to the center (death), the stronger the correlation (the higher the HR). This was scaled with the reciprocal of the HR (1/HR). Comorbidities on the line of the comorbidome were found to be non-significant in terms of all-cause mortality. A visualization supported by a comorbidome has the distinct advantage that the relevant information is readily accessible for interpretation. This applies to both, the level of prevalence and its influence on all-cause mortality.

Data analysis was performed using R Version 4.0.0 and RStudio Version 1.4. Tables, figures, and the comorbidome were created in RStudio and Microsoft Excel.

## Results

### Patient population and tumor characteristics

A total of 1745 lobectomies and segmentectomies were performed at our center between 2011 and 2020. After exclusion of re-lobectomies/-segmentectomies (*n* = 30), patients with missing information on comorbidities (*n* = 23), missing information on stage (*n* = 21), resection status (*n* = 12), and survival data (*n* = 1), the analysis was conducted on the data of 1,658 patients. Of the patients included in the study, 556 were assigned to the VATS-group and 1102 to the thoracotomy-group. The BMI-values were significantly higher in the thoracotomy-group compared to the VATS (26.2 versus 25.5, *p* = 0.002). Similarly, a significant difference was observed in tumor size, with the thoracotomy group exhibiting larger tumors (4.0 versus 2.5, *p* < 0.0001). The proportion of females in the VATS-group was significantly higher compared to the thoracotomy-group (58.6% versus 39.5, *p* < 0.0001). The median CCI of the VATS and thoracotomy group was 3.0, indicating that there were no significant differences. Patients with a PAD had median CCI of 5.0 and patients with a CAD 4.0. In the thoracotomy cohort, patients with myocardial infarction (MI) exhibited a Charlson Comorbidity Index (CCI) of 4.0. In contrast, the VATS group demonstrated a CCI of 5.0.The histopathological results after resection showed significant variations in the distribution of pN stages and resection status between the two groups. The proportion of pN0 was higher in the VATS-group than in the thoracotomy group (85.4% vs. 65.2%), whereas the proportion of pN1 (7.9% vs. 16.0%) and pN2 (6.7% vs. 18.9%) was lower (*p* < 0.0001). The proportion of R0 was higher in the VATS-group than in the thoracotomy-group (99.6% vs. 97.5%), whereas the proportion of R1 (0.4% vs. 2.3%) and R2 (0.0% vs. 0.2%) was lower (*p* = 0.003). The data indicates that 59.5% (*n* = 987) of the patients had adenocarcinoma, making it the most common tumor type histologically, followed by squamous cell carcinoma with 24.1% (*n* = 399). The distribution of histology was found to vary significantly between the two groups (*p* < 0.0001). The patient and tumor characteristics are summarized in Table 1.

**Table 1:** Patient and tumor characteristics of the study population

	All patients (*n* = 1 658)		VATS (*n* = 556)		Thoracotomy (*n* = 1 102)		
	Mean	sd	Mean	sd	Mean	sd	p-value
Age in years	65.9	10.5	66.5	10.6	65.7	10.5	0.42
BMI	26.0	4.8	25.5	4.76	26.2	4.82	0.002
Tumor size in cm	3.5	2.4	2.5	1.3	4.0	2.7	< 0.0001
	n	%	n	%	n	%	p-value
**Sex**							
Female	761	45.9%	326	58.6%	435	39.5%	
Male	897	54.1%	230	41.4%	667	60.5%	< 0.0001
pN stage							
pN0	1193	72.0%	475	85.4%	718	65.2%	
pN1	220	13.3%	44	7.9%	176	16.0%	
pN2	245	14.8%	37	6.7%	208	18.9%	< 0.0001
**M stage**							
M0	1589	95.8%	541	97.3%	1048	95.1%	
M1a	20	1.2%	5	0.9%	15	1.4%	
M1b	52	3.1%	9	1.6%	43	3.9%	
M1c	6	0.4%	1	0.2%	5	0.5%	0.21
**R stage**							
R0	1629	98.3%	554	99.6%	1075	97.5%	
R1	27	1.6%	2	0.4%	25	2.3%	
R2	2	0.1%	0	0.0%	2	0.2%	0.003
Histological type							
Adenocarcinoma	987	59.5%	392	70.5%	595	54.0%	
Squamous-cell carcinoma	399	24.1%	84	15.1%	315	28.6%	
Other histological type	272	16.4%	80	14.4%	192	17.4%	< 0.0001

Patient and tumor characteristics of lung cancer patients with lobectomy/segmentectomy stratified by surgical approach. Means with standard deviation of numerical variables and absolute and relative frequency of categorical variables

Sd = standard deviation, BMI = body mass index, pN = pathological lymph node stage, M = metastasis, R = residual tumor

### Comorbidities

As demonstrated in Table 2, the prevalence of comorbidities is evident. The most prevalent comorbidities were arterial hypertension (53.7%, *n* = 891), followed by COPD (30.7%, *n* = 509) and coronary heart disease (15.6%, *n* = 259). The following comorbidities also had a prevalence of over 5% in the patient population: diabetes, atrial fibrillation, PAD, MI, CVD and asthma.

**Table 2:** Comorbidities and prevalence of the patient population

	All patients (*n* = 1 658)		VATS (*n* = 556)		Thoracotomy (*n* = 1 102)		*p*-value
	n	%	n	%	n	%	
Myocardial infarction	89	5.4%	26	4.7%	63	5.7%	0.44
Diabetes	209	12.6%	62	11.2%	147	13.3%	0.23
Diabetes with complication	26	1.6%	6	1.1%	20	1.8%	0.35
Peripheral artery disease	127	7.7%	38	6.8%	89	8.1%	0.42
Cerebro vascular disease	88	5.3%	32	5.8%	56	5.1%	0.64
Dementia	3	0.2%	1	0.2%	2	0.2%	1.00
Asthma	88	5.3%	40	7.2%	48	4.4%	0.02
Peptic ulcer disease	35	2.1%	13	2.3%	22	2.0%	0.78
Mild liver disease	28	1.7%	10	1.8%	18	1.6%	0.96
Hemi-/paraplegia	1	0.1%	0	0.0%	1	0.1%	1.00
Moderate to severe liver disease	15	0.9%	3	0.5%	12	1.1%	0.40
Renal insufficiency	67	4.0%	21	3.8%	46	4.2%	0.80
Chronic lymphatic leukemia	4	0.2%	3	0.5%	1	0.1%	0.11
Lymphoma	20	1.2%	12	2.2%	8	0.7%	0.02
Metastatic cancer	18	1.1%	7	1.3%	11	1.0%	0.82
Coronary heart disease	259	15.6%	63	11.3%	196	17.8%	< 0.001
Atrial fibrillation	132	8.0%	36	6.5%	96	8.7%	0.14
Arterial hypertension	891	53.7%	302	54.3%	589	53.4%	0.78
Pulmonary hypertension	16	1.0%	3	0.5%	13	1.2%	0.32
Fibrosis	20	1.2%	3	0.5%	17	1.5%	0.13
NYHA	18	1.1%	2	0.4%	16	1.5%	0.08
COPD	509	30.7%	155	27.9%	354	32.1%	0.09

Absolute and relative frequency of comorbidities and prevalence by surgical approach

Sd = standard deviation, NYHA = New York Heart Association, COPD = chronic obstructive pulmonary disease

A total of 838 comorbidity occurrences were observed in the VATS-group. The prevalence of the most common comorbidities mirrors the overall distribution of the cohort. Arterial hypertension was the most common comorbidity with 54.3% (*n* = 302), followed by COPD (27.9%, *n* = 155) and CAD (11.3%, *n* = 63). Diabetes, asthma, PAD, arterial fibrillation and CVD also had prevalence rates above the 5% mark.

1825 comorbidity occurrences were observed in patients with thoracotomy. The ranking within the thoracotomy-group is consistent with the ranking within the overall cohort, with the exception of asthma, which no longer reaches a 5% prevalence. The most common comorbidity, arterial hypertension, was identified with 53.4% (*n* = 589) of the patients, COPD 32.1% (*n* = 354) and coronary heart disease 17.8% (*n* = 196). Asthma was significantly more common in the VATS-group (7.2% versus 4.4%, *p* = 0.02), so was lymphoma (2.2% versus 0.7%, *p* = 0.02). A statistically significant discrepancy was identified between the two groups in terms of the prevalence of coronary heart diseases. The thoracotomy group exhibited a higher incidence of coronary heart diseases compared to the VATS group, with a percentage of 17.8% versus 11.3%, respectively. This difference was found to be statistically significant (p = < 0.001).

### Comorbidome

**Fig. 1:**
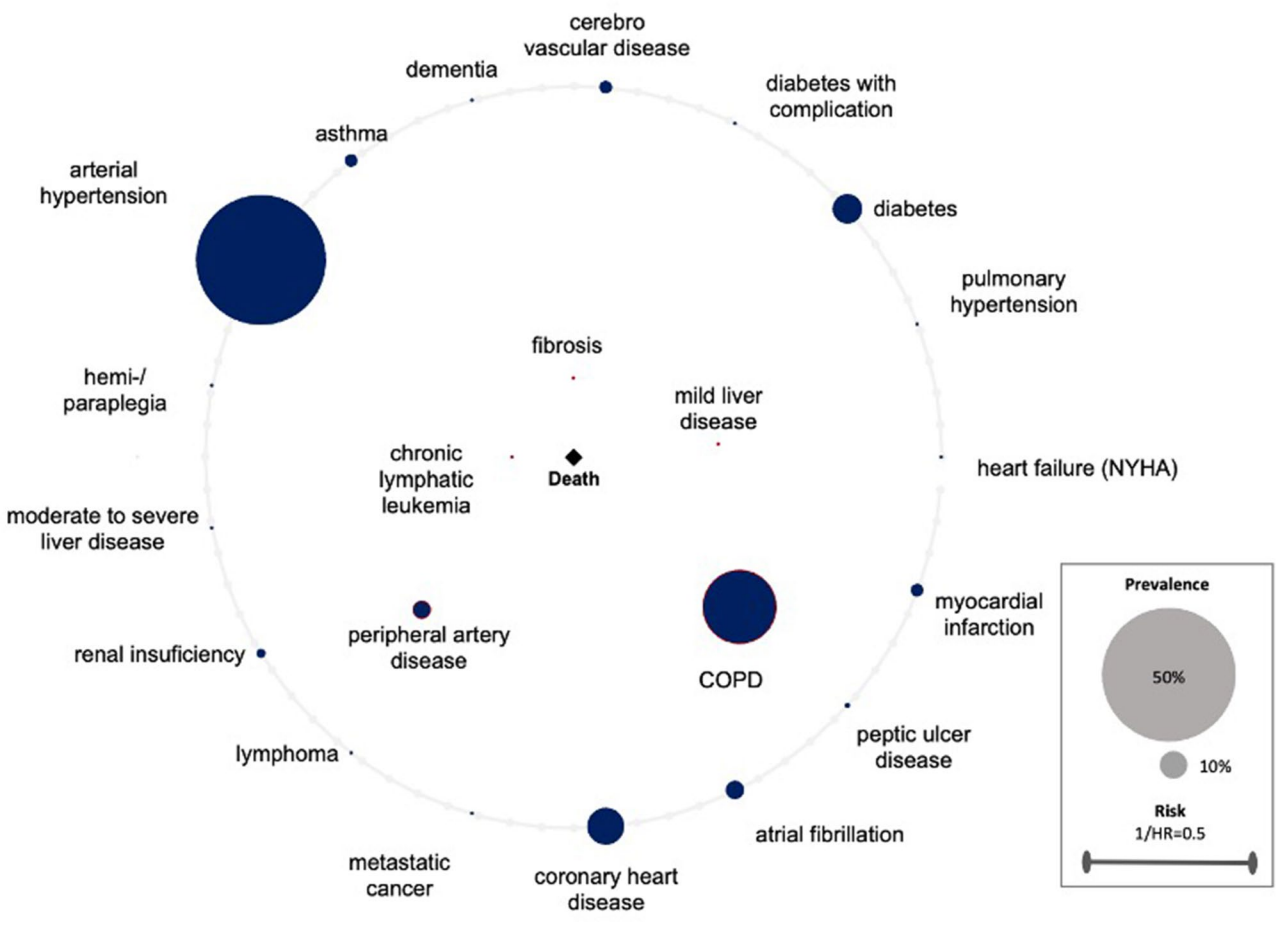
All patients. Comorbidome of surgically treated lung cancer patients displaying the association of comorbidities and all-cause mortality risk. The radius of the individual comorbidity dots reflects the prevalence of comorbidities. Dots within the comorbidome circle had a significant influence on all-cause mortality. The closer they were to the center (death), the stronger is the association to all-cause mortality (the higher the HR). This was scaled from the inverse of the HR (1/HR). Comorbidities on the line of the comorbidome had no significant influence on all-cause mortality. HR = hazard ratio, COPD = chronic obstructive pulmonary disease, MI = myocardial infarction

The application of the Cox regression model to the entire population, revealed five comorbidities with a significant impact on all-cause mortality. The comorbidity with the strongest association to all-cause mortality was chronic lymphatic leukemia (HR = 5.15, p = < 0.001), followed by fibrosis (HR = 4.06,p = < 0.001), mild liver disease (HR = 2.18, *p* = 0.02), PAD (HR = 1.48, *p* = 0.04) and COPD (HR = 1.42, p = < 0.01). The statistical evaluation can be found in the 1st column of Table 3. The graphical representation in Fig. 1. The model demonstrated a concordance level of 0.68, with a standard error of 0.02. (Table 3; Fig. 1)

**Table 3:** Results from Cox regression analysis of comorbidities and all-cause mortality risk

	All patients (*n* = 1 658)		VATS (*n* = 556)		Thoracotomy (*n* = 1 102)	
	HR	p-value	HR	p-value	HR	p-value
Myocardial infarction	1.21	0.42	2.45	0.04	0.87	0.65
Diabetes	0.88	0.47	1.07	0.84	0.75	0.18
Diabetes with complication	1.64	0.18	2.62	0.25	1.54	0.3
Peripheral artery disease	1.48	0.04	2.05	0.06	1.46	0.09
Cerebro vascular disease	0.92	0.75	1.15	0.76	0.71	0.3
Dementia	0.91	0.93			0.93	0.94
Asthma	0.71	0.27	0.47	0.28	0.77	0.49
Peptic ulcer disease	0.85	0.66	0.43	0.42	1.1	0.81
Mild liver disease	2.18	0.02	5.01	0.01	1.9	0.12
Hemi-/paraplegia	2.06	0.48			1.78	0.57
Moderate to severe liver disease	1.24	0.67			1.53	0.41
Renal insuficiency	1.24	0.32	1.31	0.54	1.18	0.52
Chronic lymphatic leukemia	5.15	< 0.001	14.31	0.01		
Lymphoma	0.87	0.79	0.89	0.91	0.65	0.56
Metastatic cancer	0.67	0.5			0.54	0.54
Coronary heart disease	1.16	0.33	1.03	0.95	1.16	0.4
Atrial fibrillation	0.96	0.83	1.01	0.98	0.93	0.75
Arterial hypertension	1.06	0.63	1.07	0.8	1.05	0.73
Pulmonaery hypertension	0.55	0.32			0.73	0.6
Fibrosis	4.06	< 0.001			4.2	< 0.001
NYHA	0.96	0.93			1.67	0.35
COPD	1.42	< 0.01	1.22	0.45	1.51	< 0.01

Results from multivariate Cox regression analysis of comorbidities with statistically significant risk for death (HR > 1), adjusted by age, sex, tumor size in cm, lymph node involvement (pN), metastases status, (cM), resection status (R), and histological type

HR = hazard ratio, NYHA = New York Heart Association, COPD = chronic obstructive pulmonary disease

**Fig. 2:**
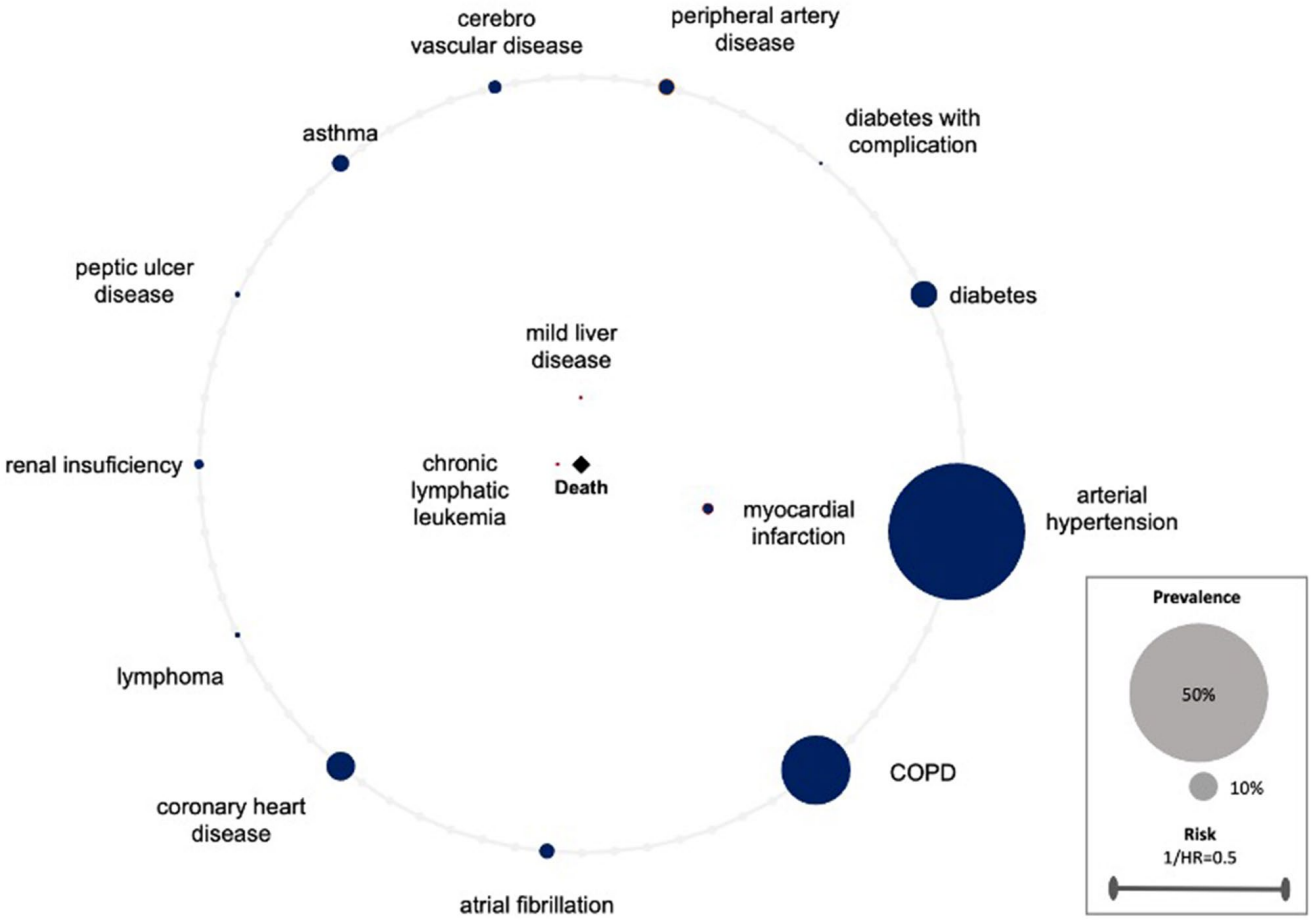
VATS group. Comorbidome of surgically treated lung cancer patients displaying the association of comorbidities and all-cause mortality risk. The radius of the individual comorbidity dots reflects the prevalence of comorbidities. Dots within the comorbidome circle had a significant influence on all-cause mortality. The closer they were to the center (death), the stronger is the association to all-cause mortality (the higher the HR). This was scaled from the inverse of the HR (1/HR). Comorbidities on the line of the comorbidome had no significant influence on all-cause mortality. HR = hazard ratio, COPD = chronic obstructive pulmonary disease, MI = myocardial infarction

In contrast to the overall cohort, only three comorbidities in the VATS-group exhibited a significant impact on all-cause mortality in operated NSCLC patients. As in the overall population, chronic lymphatic leukemia demonstrated the strongest association with all-cause mortality (HR = 14.31, *p* = 0.01). Subsequently, the occurrence of mild liver disease (HR = 5.01,*p* = 0.01) and MI (HR = 2.45, *p* = 0.04) were observed. PAD was no longer found to be statistically significant with a p-value of 0.06. The data can be found in Table 3, 2nd column, the comorbidome for VATS segmentectomy/lobectomy is shown in Fig. 2.

**Fig. 3:**
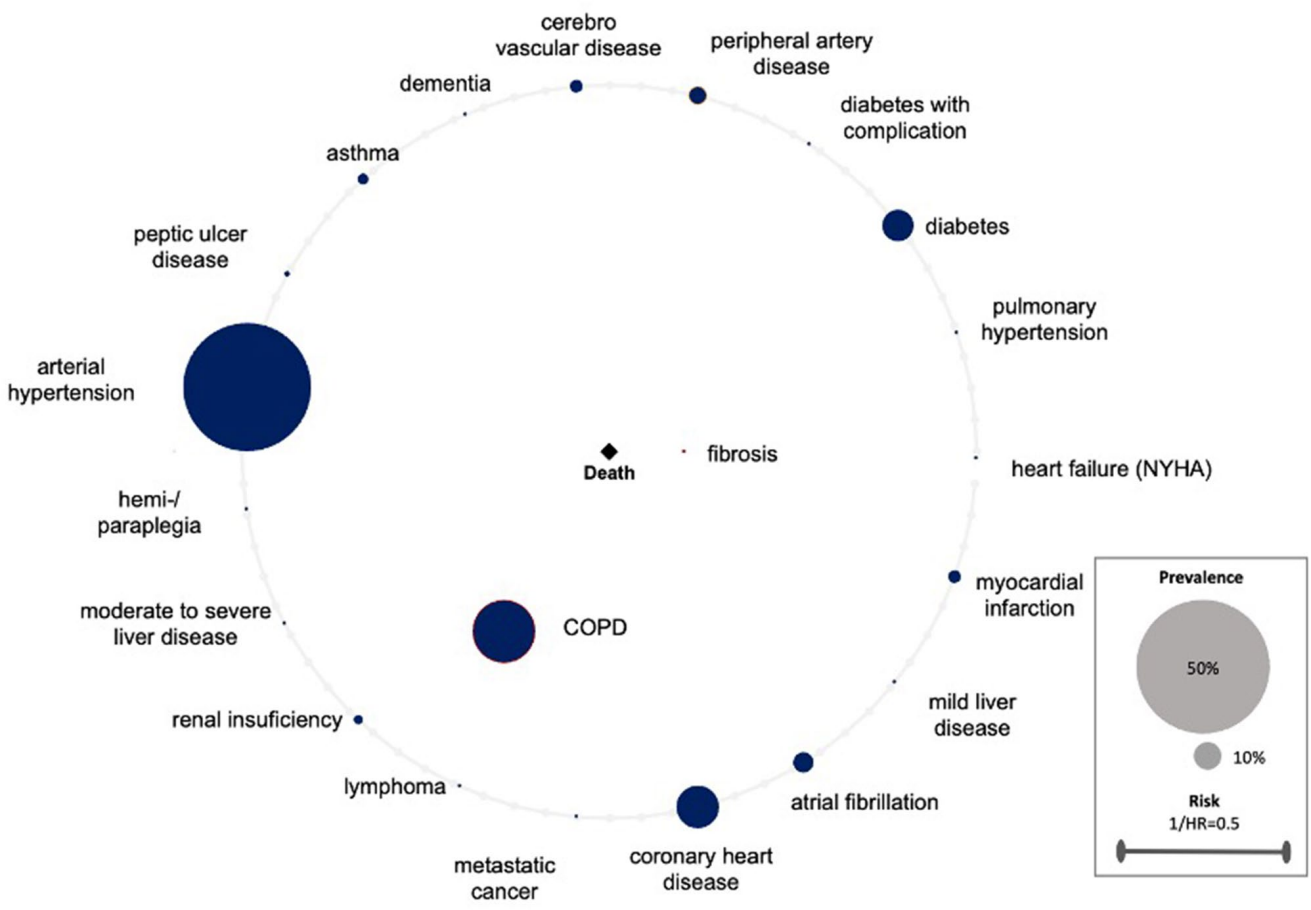
Thoracotomy group. Comorbidome of surgically treated lung cancer patients displaying the association of comorbidities and all-cause mortality risk. The radius of the individual comorbidity dots reflects the prevalence of comorbidities. Dots within the comorbidome circle had a significant influence on mortality. The closer they were to the center (death), the stronger is the association to all-cause mortality (the higher the HR). This was scaled from the inverse of the HR (1/HR). Comorbidities on the line of the comorbidome had no significant influence on all-cause mortality. HR = hazard ratio, COPD = chronic obstructive pulmonary disease, MI = myocardial infarction

A subsequent investigation of patients undergoing thoracotomy revealed that two comorbidities had a significant influence on all-cause mortality (Table 3rd column and Fig. 3). The strongest correlation was identified for fibrosis (HR = 4.20,p = < 0.001). In addition to fibrosis, COPD was found within the comorbidome circle (HR = 1.51,p = < 0.01). As was the case in the VATS-group, PAD was not identified as significant in the thoracotomy-group (*p* = 0.09). The C-index of this model was 0.70 with a standard error of 0.03.

A meticulous examination of the cardiopulmonary risk factors revealed that COPD and fibrosis (lung disease) were significant contributors to all-cause mortality in the thoracotomy group. In contrast, MI (heart disease) emerged as a significant factor in the VATS group. The model demonstrated a concordance level of 0.68, with a standard error of 0.02.

## Discussion

This present study comprised 1658 NSCLC patients who underwent lobectomy or segmentectomy at our Lung Cancer Center. Accordingly, the present study constitutes one of the most substantial single-center publications to date. We were able to identify preoperative comorbidities with an impact to all-cause mortality of lung cancer patients undergoing surgery and represented those in a comorbidome. Beside the overall evaluation of the total study population, we specifically compared the preferred operation techniques, VATS and thoracotomy.

The application of comorbidity measures is frequently observed in the context of internistic studies. In particular COPD-comorbidomes and pulmorbidomes are being used to denote risk factors for elevated mortality rates with COPD-patients or patients having an interstitial lung disease [14–16]. 

We are under the assumption that we are the first surgical study group to utilize comorbidomes to graphically present comorbidities with an impact to all-cause mortality of lung cancer patients undergoing surgery. Murawski et al. previously included lung cancer patients undergoing surgery in Germany as part of their analysis. However, the data utilized in their study originated from a nationwide statutory health insurance query [17]. Therefore, it is hypothesized that the data we used in our study are more precise because they were derived from our electronic patient records and patient archives, thus excluding errors and missing data that may occur during data transmissions to health insurance companies.

Our analysis showed that for both, the cohort as a whole and for the two subgroups, the comorbidities exhibiting the most significant impact on all-cause mortality have a low prevalence (Tables 2 and 3). Compared to the current literature, the prevalences of our study group are representative. A large study encompassing 16,367 chronic lymphatic leukemia (CLL) patients revealed that 2.57% of the patients developed lung cancer [18]. It is noteworthy that, in general, merely approx. 20–25% of lung cancer patients are at an operable stage at the time of diagnosis [19]. This figure is further reduced in the presence of concomitant oncological diseases, such as chronic lymphatic leukemia. With that, a prevalence of 0,2% of operated chronic lymphocytic leukemia patients is comprehensible. Solomon et al. demonstrated that lung cancer patients with CLL have a lower overall survival rate compared to those who do not have CLL [20]. We were able to demonstrate that this also applies to lung cancer patients who have undergone surgery. Chronic lymphatic leukemia exhibited the most significant influence in our analyses, likely attributable to the concurrence of double tumors and compromised immune function.

Pulmonary fibrosis has been determined to be the second strongest influence in our present analysis. Lung resection is only rarely indicated for patients with pulmonary fibrosis. Their lung function is so severely impaired that the feasibility of surgical intervention is precluded. This is also the reason for the low prevalence of 1.2%. In instances where lung function is within an operable range, they still have a very high mortality risk for surgery, in particular due to a postoperative acute exacerbation [21, 22]. The underlying causes of this phenomenon are not fully understood. However, it is assumed that, on the one hand, hyperoxia produces free radicals which are believed to exacerbate the severity of the outcome and, on the other hand, lung damage is caused by mechanical ventilation [23]. Even in our high volume center, only a few patients with known pulmonary fibrosis are operated on. Beyond the finding that fibrosis constitutes the second highest mortality risk within the overall cohort, our results revealed that fibrosis is identified as the highest all-cause mortality risk within the thoracotomy group. (Table 3; Figs. 1 and 2) This case exemplifies the potential risks associated with surgical intervention in patients diagnosed with pulmonary fibrosis. In addition to the targeted selection of these patients for surgical treatment, close postoperative monitoring is imperative. Postoperative pneumonia can be detected by blood tests and radiological follow-up. Patients should undergo intensive respiratory training to mitigate the risk of complications. Prophylactic perioperative antibiotics may also be considered. We recommend that these patients receive treatment in experienced centers that offer comprehensive intensive medical care and are closely monitored as outpatients.

MLD has a very low prevalence but a high all-cause mortality risk. As illustrated in Table 3, the mortality risk is elevated among patients with liver disease who have undergone surgical intervention, irrespective of the surgical approach [24]. In this context, it is comprehensible that the all-cause mortality among lung cancer patients who have undergone surgery is also increasing. In order to be able to better estimate the surgical risk, it may be beneficial to calculate the Child-Pugh-Score again preoperatively. This could potentially mitigate the progression of liver damage from a more severe state.

Comorbidities, which are prevalent and significantly influence the all-cause mortality of operated patients, primarily affect the cardiovascular and pulmonary systems. PAD is only showing a significant all-cause mortality risk within the overall population; in the subgroups there is barely more risk. (Table 3) It is known, that patients having PAD and CAD do generally have an increased risk of ischemic stroke, MI and cardiovascular death [25]. 

However, we were surprised to see that, in our study, PAD showed an increased all-cause mortality risk, while CAD did not. Consequently, a more thorough examination of these patients was undertaken. We determined that 44% of patients diagnosed with PAD also suffered from CAD, but only 22% of patients with CAD also had PAD. The extant literature describes that a combination of PAD and CAD significantly increases the risk of mortality [26, 27]. As the combination of PAD and CAD is more common in the PAD group, this observation could serve as a potential explanation for the augmented risk observed in our analysis. Furthermore, these findings underscore the heightened risk of all-cause mortality associated with the combination of PAD and CAD in surgical lung cancer patients. Additionally we took a closer look at on the CCI for PAD- and CAD patients. Patients with a documented PAD had a median CCI of 5.0, patients with a documented CAD only 4.0. Consequently, the observed performance of the PAD-index in our analysis could be attributable not only to the combination with other comorbidities such as CAD, but also due to the fact that patients with PAD exhibited a higher degree of overall illness severity prior to surgery. In order to reduce the risk of mortality, extended cardiologic diagnostics could be helpful in these patients preoperatively.

Additional disparities were found across the subgroups. Patients who underwent VATS surgery and had a history of MI exhibited a significantly higher all-cause mortality rate. (Fig. 2) It is known that MI is one of the major postoperative complications [28]. However, Takenaka et al. reported that preoperative cardiovascular comorbidities don’t have any influence on the operative outcome [29]. The reasons for the elevated all-cause mortality observed in patients with prior MI and subsequent lung surgery, particularly the significant increase seen in the VATS group compared to the thoracotomy group, remain unclear. As a minimally invasive procedure, VATS is a tissue-preserving surgical procedure that results in shorter hospital stays, less pain and is suitable for patients with comorbidities due to its low invasiveness [30–32]. Therefore, we took a closer look at the patients with MI, considering that the patients in the VATS group may have had more comorbidities compared to the thoracotomy group and may have been multimorbid. This finding was confirmed with a median CCI of 5.0 in the VATS group having MI compared to a median CCI of 4.0 in the thoracotomy group with MI. The VATS patients with documented MI were slightly sicker prior to undergoing surgery when compared with the thoracotomy patients. However, further prospective studies are necessary to thoroughly investigate the relationship between VATS surgery for lung cancer and pre-existing MI.

In the thoracotomy group, COPD was associated with a significantly increased rate of all-cause mortality. (Table 3; Fig. 2) In our opinion, COPD as a risk factor can be explained as follows: The risk of all-cause mortality is significantly increased in COPD patients due to an acute exacerbation. Patients who undergo thoracotomy are more prone to acute exacerbation, as the procedure results in greater tissue damage when compared with VATS. This results in more postoperative pain and more pulmonary complications such as pneumonia [31–33]. Pneumonia and respiratory tract infections are the primary cause of acute exacerbation of COPD [34]. COPD accounts for a large proportion of our patients, with a prevalence of 30.7%. Of all comorbidities that have a significant impact on all-cause mortality, it is the one with the highest prevalence. Given the frequency of these events, further studies are necessary to determine whether the all-cause mortality risk in COPD patients is primarily triggered by acute exacerbations, which can be prevented if necessary, or whether there are other contributing factors. In order to mitigate the risk of all-cause mortality, it is recommended that patients with known COPD undergo surgery using VATS rather than thoracotomy in large centers with sufficient expertise in lung surgery. It should also be clarified preoperatively whether the COPD is well controlled with medication. Conversely, the condition bears resemblance to pulmonary fibrosis in that pneumonia should be identified promptly. Prophylactic antibiotics may be beneficial, and the patient should undergo intensive breathing training.

Arterial hypertension, which demonstrated the highest prevalence among all groups, had no influence on all-cause mortality. Similarly, the previously described CAD, which demonstrated the third highest prevalence among all groups, did not exert any influence on all-cause mortality.

The present study is not without its limitations. Firstly, it should be noted that this is a retrospective single center study. Secondly, the analyses indicated that it is important to consider the comorbidities individually, while also acknowledging the potential impact of combinations of comorbidities on all-cause mortality risk. These high-risk comorbidity combinations should be identified in subsequent studies. This will allow for adequate preparation for major lung surgery.

## Conclusion

In summary, it is fortunate that the majority of comorbidities do not have a significant impact on postoperative all-cause mortality in lung surgery. Conversely, those with a high impact generally exhibit a very low prevalence. For patients suffering from pulmonary fibrosis or liver disease the all-cause mortality risk needs to be taken into account. PAD poses a significant risk, particularly in conjunction with CAD, and the frequent multimorbidity of these patients must not be disregarded. Particularly important is COPD, which has a significantly increased all-cause mortality risk, with a prevalence of 30.7% of all patients. Ideally, these patients should undergo minimally invasive surgery in a high-volume center with a high degree of expertise. Further studies are necessary to elucidate the precise factors contributing to the suboptimal outcomes observed in patients with COPD following thoracotomy for lung cancer surgery.

## Data Availability

The datasets and materials of the current study are available from the corresponding author upon reasonable request.Data from 1658 patients were recorded in the database. This data is still being analyzed and further studies are in progress. In order not to jeopardize the data for the new studies, they cannot be disclosed at this time. We ask for your understanding.

## Abbreviations

ACC: Adenocarcinoma
BMI: Body Mass Index
CAD: Coronary heart disease
CCI: Charlson-Comorbidity-Index
CLL: Chronic Lymphatic Leukemia
cM: Metastases status
COPD: Chronic Obstructive Pulmonary Disease
CVD: Cerebro Vascular Disease
HR: Hazard Ratio
MI: Myocardial Infarction
MLD: Mild Liver Disease
NSCLC: Non-Small Cell Lung Cancer
NYHA: New York Heart Association
PAD: Peripheral Artery Disease
pN: lymph node involvement
R: Resection status
SCC: Squamous-Cell Carcinoma
SD: Standard Deviation
VATS: Video-Assisted Thoracoscopic Surgery
